# Prevalence of Dyslipidemia in Patients Receiving Health Checkups: A Hospital-Based Study

**DOI:** 10.1155/2011/314234

**Published:** 2011-10-26

**Authors:** Kao-Chi Cheng, Yu-Lung Chen, Shih-Wei Lai

**Affiliations:** ^1^School of Medicine, China Medical University, Taichung 40447, Taiwan; ^2^Department of Family Medicine, China Medical University Hospital, Taichung 40447, Taiwan

## Abstract

We used the dataset from one medical center in Taiwan to explore the prevalence of dyslipidemia, which included 2695 subjects receiving private health checkups in 2003-2004. The overall prevalence of hypercholesterolemia was 53.3% in men and 48.2% in women (*P* = 0.008). The overall prevalence of hypertriglyceridemia was 29.3% in men and 13.7% in women (*P* < 0.001). The overall prevalence of elevated LDL level was 50.7% in men and 37.9% in women (*P* < 0.001). The overall prevalence of low HDL level was 47.4% in men and 53% in women (*P* = 0.004).

## 

We analyzed 2695 subjects who received private health checkups at their own expense at one medical center located at the Taichung city in mid-Taiwan from 2003 to 2004. There were 1526 men (56.6%) and 1169 women (43.4%). The mean age was 49.2 years old (standard deviation 12.2, range from 20 to 84). Hypercholesterolemia was defined as total cholesterol level ≥200 mg/dL [1]. Hypertriglyceridemia was defined as triglyceride level ≥150 mg/dL [2]. Elevated low-density lipoprotein cholesterol (LDL) level was defined as LDL level ≥130 mg/dL [2]. Low level of high-density lipoprotein cholesterol (HDL) was defined as HDL <40 mg/dL in men and <50 mg/dL in women [2].

In Figure 1, the overall prevalence of hypercholesterolemia was 53.3% in men and 48.2% in women (*P *= 0.008). In the group aged 20 to 39, men had higher prevalence of hypercholesterolemia than women did (48.2% versus 22.6%, *P * < 0.001). In the group aged 65 and over, women had higher prevalence than men did (63.5% versus 47.2%, *P *= 0.003). There was no significant difference between men and women in the group aged 40 to 64 (55.9% versus 53.8%, *P *= 0.368). The prevalence of hypercholesterolemia increased with age in women (*P * < 0.001).

In Figure 2, the overall prevalence of hypertriglyceridemia was 29.3% in men and 13.7% in women (*P * < 0.001). Men had higher prevalence of hypertriglyceridemia than women did in the group aged 20 to 39 and in the group aged 40 to 64 (*P * < 0.001 and *P * < 0.001, resp.). There was no significant difference between men and women in the group aged 65 and over (18.2% versus 26.4%, *P *= 0.077). The prevalence of hypertriglyceridemia increased with age in women (*P * < 0.001).

In Figure 3, the overall prevalence of elevated LDL level was 50.7% in men and 37.9% in women (*P * < 0.001). Men had higher prevalence of elevated LDL level than women did in the group aged 20 to 39 and in the group aged 40 to 64 (*P * < 0.001 and *P*<0.001, resp.). There was no significant difference between men and women in the group aged 65 and over (46.0% versus 48.6%, *P *= 0.637). The prevalence of elevated LDL level increased with age in women (*P * < 0.001).

In Figure 4, the overall prevalence of low HDL level was 47.4% in men and 53% in women (*P *= 0.004). Women had higher prevalence of low HDL level than men did in the group aged 40 to 64 and in the group aged 65 and over (*P *= 0.047 and *P *= 0.001, resp.). There was no significant difference between men and women in the group aged 20 to 39 (46.3% versus 47.1%, *P *= 0.857). The prevalence of low HDL level increased with age in women (*P *= 0.014). 

In 2010, cardiovascular disease and cerebrovascular disease are the second and the third leading causes of death in Taiwan [3]. Previous reports have suggested dyslipidemia, including hypercholesterolemia (elevated total cholesterol level), hypertriglyceridemia, elevated LDL level, and low HDL level, is a major risk factor for cardiovascular disease and ischemic stroke [4–6]. In this present study, the overall prevalence of dyslipidemia is relatively high, including hypercholesterolemia 51.1%, hypertriglyceridemia 22.5%, elevated LDL level 45.1%, and low HDL level 49.8%. In addition, the prevalence rates of hypercholesterolemia, hypertriglyceridemia, elevated LDL level, and low HDL level significantly increase with age in women. That is, the prevalence of dyslipidemia in women is lower in the group aged 20–39 than in the group aged 40 and over, but no similar trend is found in men. This result further reveals that the status of all serum lipids in women aged 20–39 is significantly better than the other age groups. However, in this dataset, there was only blood data, no mention whether these patients were using lipid-lowering drugs. Thus, the prevalence of dyslipidemia might be underestimated. Meanwhile, we hope these findings are to guide public health interventions to manage dyslipidemia issue in Taiwan.

## Figures and Tables

**Figure 1:**
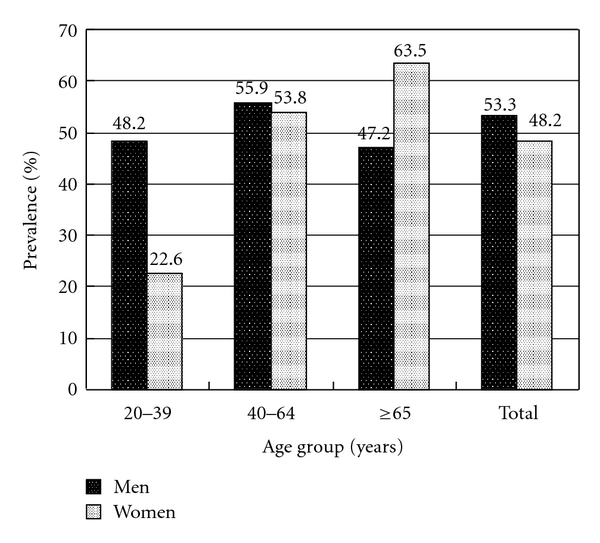
Prevalence of hypercholesterolemia in gender and three age groups.

**Figure 2:**
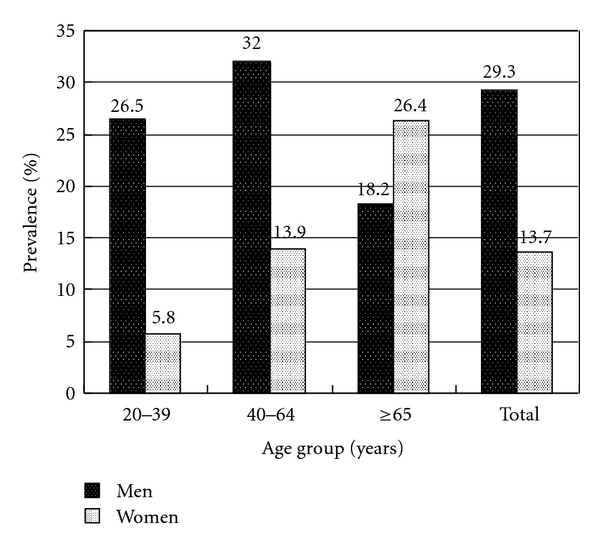
Prevalence of hypertriglyceridemia in gender and three age groups.

**Figure 3:**
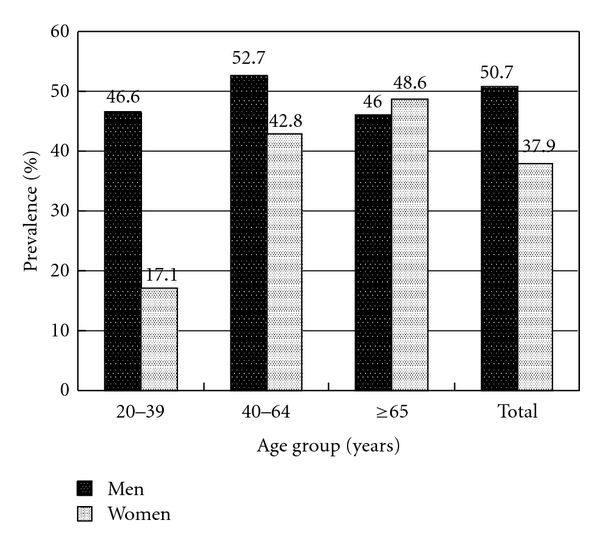
Prevalence of elevated LDL level in gender and three age groups.

**Figure 4:**
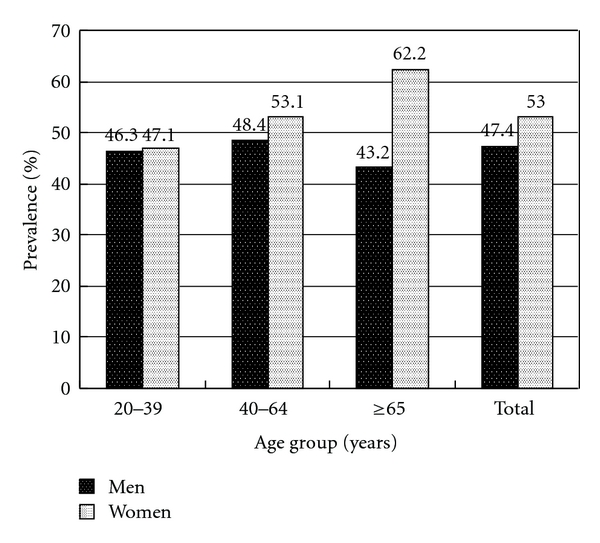
Prevalence of low HDL level in gender and three age groups.
